# Optic nerve elasticity and sheath diameter in amblyopia: A cross-sectional study

**DOI:** 10.1097/MD.0000000000049801

**Published:** 2026-07-17

**Authors:** Behice Kaniye Yilmaz, Aynur Diracoglu, Sevim Ozdemir, Tuba Selcuk Can

**Affiliations:** a Department of Radiology, Kanuni Sultan Süleyman Training and Research Hospital, Istanbul, Türkiye; b Department of Ophthalmology, Haseki Training and Research Hospital, Istanbul, Türkiye; c Department of Radiology, Haseki Training and Research Hospital, Istanbul, Türkiye.

**Keywords:** amblyopia, optic nerve elasticity, optic nerve sheath diameter, shear-wave elastography

## Abstract

This study aimed to examine and compare optic nerve elasticity (ONE) and optic nerve sheath diameter (ONSD) in amblyopic eyes and healthy fellow eyes. This prospective, observational study was conducted between October 2019 and December 2019. A total of 21 patients diagnosed with unilateral amblyopia were included in the study. The ONSD of both eyes of each patient was measured by ultrasonography, and ONE was measured by shear-wave elastography. The results were compared between healthy eyes and amblyopic eyes. Eleven female and 10 male patients were included in the study, and the median age of the patients was 25 (interquartile range [IQR]: 20–31) years. The mean ONSD of amblyopic eyes (2.37 ± 0.36) was significantly lower than that of healthy eyes (2.54 ± 0.39) (*P* = .014). Although the median ONE value of the amblyopic eyes (2.73 [IQR = 1.52–3.84]) was higher than that of healthy eyes (2.63 [IQR = 1.70–4.65]), this difference was not significant (*P* = .931). Mean ONSD values were significantly lower in amblyopic eyes compared with healthy fellow eyes, whereas no significant difference was observed in ONE values between the 2 groups. These findings suggest that reduced ONSD may represent a structural alteration associated with amblyopia. However, because of the cross-sectional design and limited sample size, the biological and clinical significance of these findings remains uncertain and requires further investigation in larger prospective studies.

## 1. Introduction

Amblyopia is a neurodevelopmental disorder of the visual system characterized by unilateral or, less commonly, bilateral reduction in best-corrected visual acuity in the absence of clinically detectable structural ocular abnormalities.^[1–3]^ It results from abnormal visual input during early life and is commonly associated with visual deprivation, strabismus, anisometropia, ptosis, or high bilateral refractive errors.^[1,4]^ The reported prevalence of amblyopia ranges from 1 to 5%, making it the most common cause of visual impairment in childhood.^[5,6]^ Although treatment during the period of visual plasticity may improve visual outcomes, not all patients respond successfully to therapy, suggesting that the underlying pathophysiological mechanisms of amblyopia are not yet fully understood.^[1]^

Previous studies in both human and animal models have demonstrated structural and functional abnormalities involving the lateral geniculate nucleus and visual cortex in amblyopia.^[7,8]^ More recently, increasing attention has focused on anterior visual pathway structures, including the retina, optic disc, and choroid.^[4,9–13]^ However, findings regarding retinal alterations in amblyopia remain inconsistent.^[11,14,15]^ While some studies have reported increased retinal nerve fiber layer thickness in amblyopic eyes, others have found no significant interocular differences.^[10,11,14–16]^

Shear-wave elastography (SWE) is a quantitative imaging technique that enables noninvasive assessment of tissue stiffness and elasticity.^[17]^ In recent years, elastography has been increasingly used in the evaluation of various tissues and organs, including the lymph nodes, liver, thyroid, testis, and breast.^[18]^ Associations between optic nerve elasticity (ONE) and optic nerve sheath characteristics have been investigated in several ophthalmologic and systemic disorders. Altered ONE values have been reported in optic neuritis,^[19]^ preeclampsia,^[20]^ multiple sclerosis,^[21]^ Graves disease,^[22]^ Behçet disease,^[23]^ glaucoma,^[24]^ and migraine.^[25]^

In addition, optic nerve sheath diameter (ONSD) has been recognized as an indirect marker of intracranial pressure and has been investigated as a potential prognostic parameter in several neurological conditions.^[26,27]^ However, to the best of our knowledge, no previous study has evaluated the relationship between ONSD, ONE, and amblyopia. Investigation of these parameters may contribute to a better understanding of structural alterations associated with amblyopia.

Although amblyopia treatment is effective in many children, therapeutic response may vary among patients.^[28]^ Understanding the structural and functional factors associated with amblyopia remains important. Current clinical evaluation methods may not fully reflect subtle structural alterations associated with amblyopia.^[28]^ Quantitative assessment of ONE and ONSD may provide additional information regarding structural characteristics of the optic nerve in amblyopic patients and may contribute to a better understanding of amblyopia-related changes.

Therefore, in the present study, we aimed to evaluate ONE and ONSD in amblyopic eyes and healthy fellow eyes and to investigate their potential association with amblyopia.

## 2. Methods

### 2.1. Study design and ethics

This prospective observational cross-sectional study was conducted between October 2019 and December 2019 at the Departments of Ophthalmology and Radiology, Haseki Research and Training Hospital, University of Health Sciences, Istanbul, Türkiye. The study protocol was approved by the Ethics Committee of the University of Health Sciences (approval date: October 23, 2019; approval number: 2019/2-24). Written informed consent was obtained from all participants for study participation and use of clinical data. All procedures were conducted in accordance with the principles of the Declaration of Helsinki and its subsequent amendments.

### 2.2. Study population

A total of 21 patients with unilateral amblyopia were included in the study. Inclusion criteria were age ≥ 18 years, unilateral amblyopia, best-corrected visual acuity ≤ 0.2 in the amblyopic eye, and normal visual acuity in the fellow eye.

Clinical history was obtained from all participants, including potential causes of visual impairment such as trauma, systemic disease, febrile illness, prematurity, or ocular injury. Patients with uncertain clinical history or potential alternative causes of visual impairment were excluded. All participants underwent refractive evaluation, biomicroscopic examination, fundus examination, intraocular pressure measurement, and optic nerve and macular optical coherence tomography assessment.

Following ophthalmologic evaluation, eyes with normal visual acuity and no pathological findings were included as healthy fellow eyes, whereas eyes with reduced visual acuity without additional ocular pathology were included in the amblyopia group.

Only patients with idiopathic amblyopia were included. Idiopathic amblyopia was defined as reduced best-corrected visual acuity in 1 eye in the absence of identifiable amblyogenic factors following comprehensive ophthalmologic evaluation. Patients with recognized causes of amblyopia or conditions potentially affecting optic nerve measurements, including strabismus, significant refractive error, deprivation-related amblyopia (e.g., cataract or ptosis), macular pathology, previous ocular surgery, hypertension, diabetes mellitus, or neurological disorders such as multiple sclerosis, were excluded. None of the patients had previously received amblyopia treatment.

### 2.3. Ophthalmologic and radiologic evaluation

After recording demographic data, all eligible participants underwent detailed ophthalmologic and radiologic examinations.

### 2.4. Ophthalmological examinations

All ophthalmologic examinations were performed by the same ophthalmologist. Best-corrected visual acuity was assessed in both eyes using a Snellen chart at a testing distance of 6 meters. Visual acuity values were converted to decimal equivalents for statistical analysis.^[4]^ Decimal values were used consistently throughout the study to ensure uniform reporting of visual acuity measurements. A total of 42 eyes were evaluated and classified into 2 groups: healthy fellow eyes (control group, n = 21) and amblyopic eyes (n = 21). Only patients with normal visual acuity in the fellow eye (decimal visual acuity = 1.0) and reduced visual acuity in the amblyopic eye (decimal visual acuity ≤ 0.2) were included, consistent with the diagnostic criteria for unilateral amblyopia.

### 2.5. Radiological examinations

All ultrasonographic and elastographic examinations were performed by a single experienced radiologist with approximately 15 years of ultrasonography and 3 years of elastography experience using a standardized imaging protocol to minimize measurement variability.

Examinations were performed in the Radiology Department using an Esaote MyLab 9 ultrasound system (Esaote SpA) equipped with QElaXto Pack SWE software (EVO 3.0 version) and a 4 to 15 MHz multifrequency linear transducer. All participants were examined in the supine position with their eyes closed and were instructed to remain still during the procedure. Ultrasound gel was applied over the closed eyelids to facilitate image acquisition.

**Figure 1.**
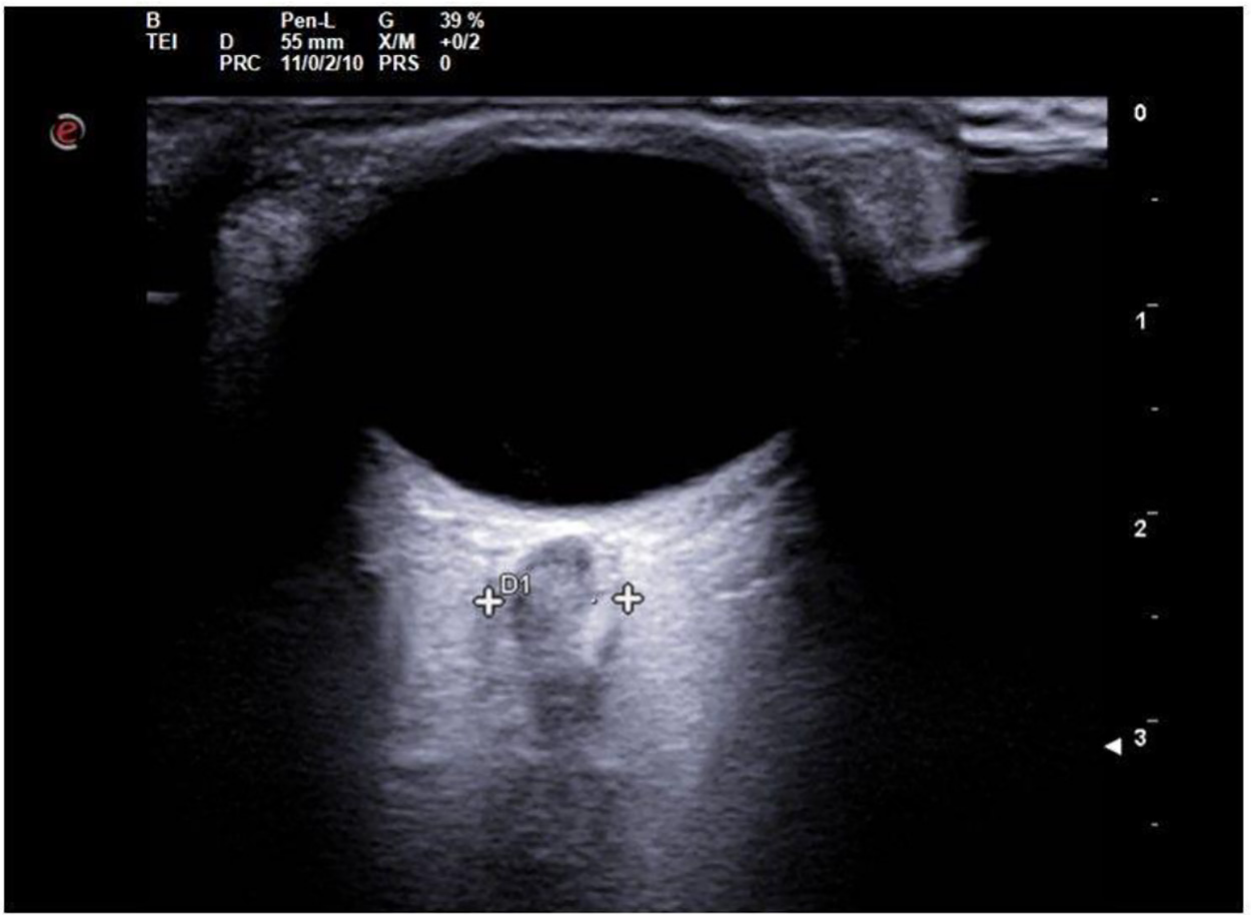
Measurement of ONSD using grayscale ultrasonography. ONSD = optic nerve sheath diameter.

During grayscale ultrasonography, ONSD measurements were obtained perpendicular to the longitudinal axis of the optic nerve between the outer hypoechoic borders. ONSD was measured 3 times in each eye at a point 3 mm posterior to the globe, and the mean value was used for analysis (Fig. 1).

**Figure 2.**
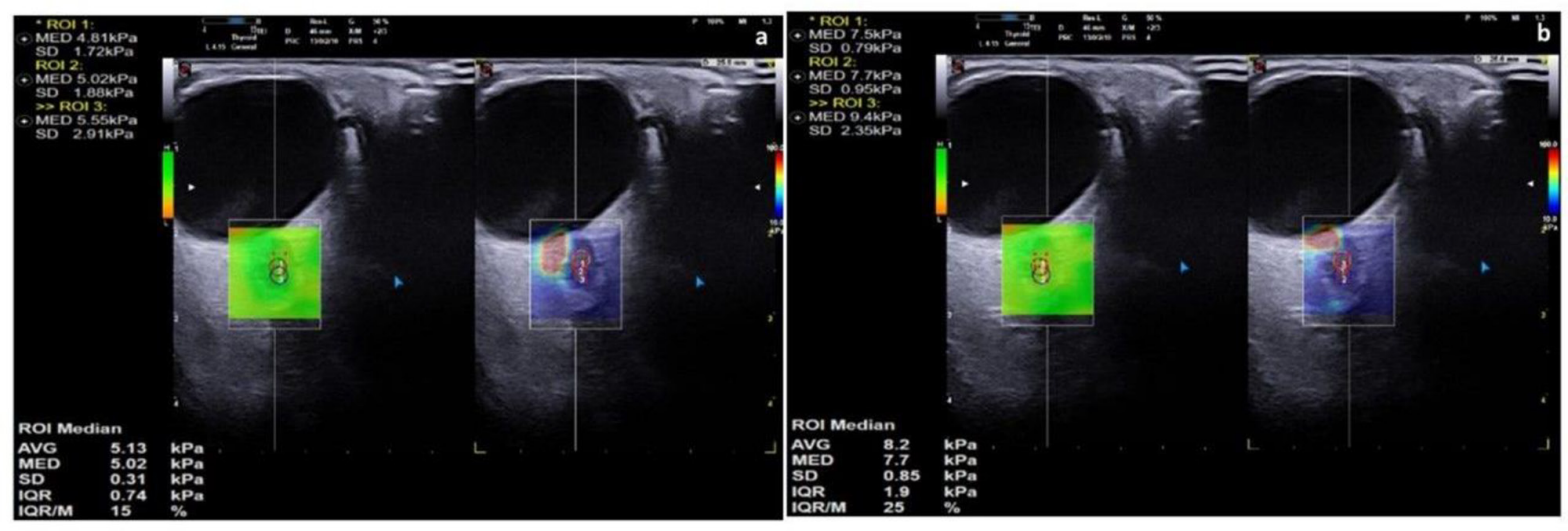
SWE measurements of the optic nerves in a 47-year-old male patient: (A) amblyopic eye and (B) healthy fellow eye. SWE = shear-wave elastography.

Subsequently, SWE measurements were obtained using a circular region of interest placed over the optic nerve. Elasticity measurements were performed 3 times in each eye and recorded in kilopascals (kPa) within a measurement range of 0 to 100 kPa, and the mean values were used for analysis. Measurements obtained from green-coded reliability regions with interquartile range (IQR)-to-median values < 30% were considered acceptable for analysis (Fig. 2).^[29]^

## 3. Statistical analysis

A priori power analysis was performed using previously published data.^[30]^ Assuming a large effect size (Cohen *d* = 0.8) for paired comparisons, the minimum required sample size was calculated as 19 participants with 95% statistical power and a significance level of 0.05 using Power Analysis and Sample Size Software (NCSS).

All statistical analyses were performed using IBM SPSS software (IBM Corp.). The Shapiro–Wilk test was used to assess data normality. Continuous variables are presented as mean ± standard deviation or median IQR according to data distribution, whereas categorical variables are presented as frequencies and percentages.

Normally distributed continuous variables were analyzed using the paired *t*-test, whereas non-normally distributed variables were analyzed using the Wilcoxon signed-rank test. Univariable linear regression analyses were performed to evaluate factors associated with differences in ONSD between amblyopic and healthy fellow eyes. A 2-tailed *P* value < .05 was considered statistically significant.

No formal adjustment for multiple comparisons was performed because the analyses were exploratory in nature.

## 4. Results

Eleven female and 10 male patients were included in the study. The median age was 25 years (IQR: 20–31; range: 18–47 years).

**Figure 3.**
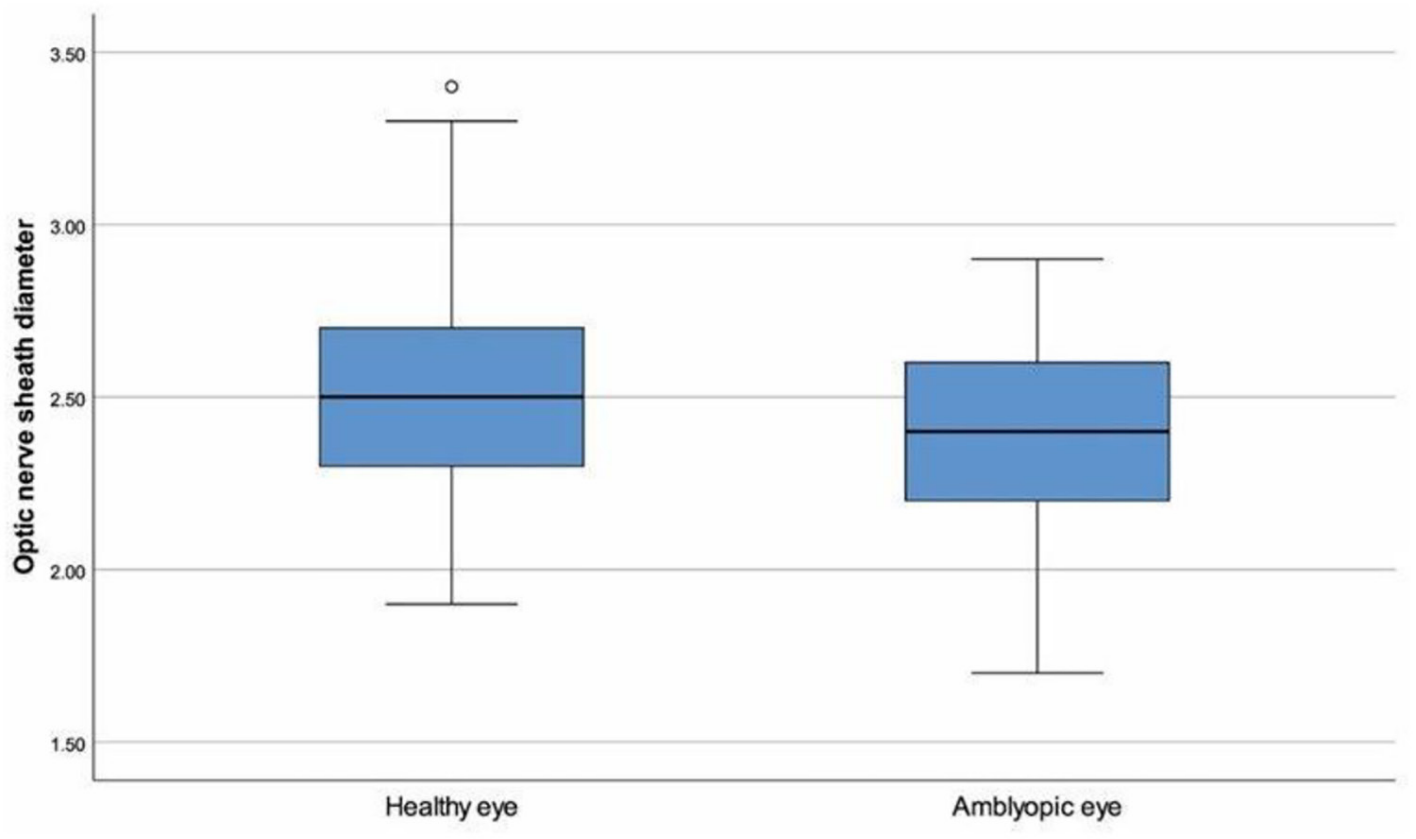
Box plots demonstrating ONSD values in amblyopic and healthy fellow eyes (*P* = .014). ONSD = optic nerve sheath diameter.

The mean ONSD in amblyopic eyes (2.37 ± 0.36 mm) was significantly lower than that in healthy fellow eyes (2.54 ± 0.39 mm), with a mean paired difference of −0.17 mm (*P* = .014). The comparison demonstrated a moderate effect size (0.584, 95% confidence interval: 0.114–1.042) (Fig. 3).

Median ONE values were similar between amblyopic eyes (2.73 kPa [IQR: 1.52–3.84]) and healthy fellow eyes (2.63 kPa [IQR: 1.70–4.65]) (*P* = .931), with a negligible effect size (−0.039, 95% confidence interval: −0.466–0.389). Patient characteristics and study findings are summarized in Table 1.

**Table 1:** Summary of patient characteristics and measurements.

Age	25 (20–31)
Sex	
Male	10 (47.6%)
Female	11 (52.4%)
Amblyopic eye	
Right	7 (33.3%)
Left	14 (66.7%)
Amblyopic eye visual acuity	0.05 (0.05–0.10)
ONSD (mm)	
Healthy eye	2.54 ± 0.39
Amblyopic eye	2.37 ± 0.36
P*	.014
Effect size (95% CI)†	0.584 (0.114–1.042)
ONE, shear-wave elastography value (kPa)	
Healthy eye	2.63 (1.70–4.65)
Amblyopic eye	2.73 (1.52–3.84)
*P*‡	.931
Effect size (95% CI)†	−0.039 (−0.466–0.389)

Data are given as mean ± standard deviation or median (1st quartile−3rd quartile) for continuous variables according to normality of distribution and as frequency (%) for categorical variables.

CI = confidence interval, ONE = optic nerve elasticity, ONSD = optic nerve sheath diameter.

* Paired *t*-test.

† Cohen *d*.

‡ Wilcoxon signed-ranks test.

According to univariable linear regression analysis, age (*P* = .341), female sex (*P* = .399), left-eye amblyopia (*P* = .601), visual acuity of the amblyopic eye (*P* = .967), and ONE values (*P* = .756) were not significantly associated with the difference in ONSD between healthy fellow eyes and amblyopic eyes (Table 2).

**Table 2:** Univariable linear regression analysis of factors associated with ONSD difference between healthy and amblyopic eyes.

	Univariable		
	Unstandardized coefficients (95% CI)	Standardized coefficients	*P*
Age	0.008 (−0.009–0.025)	0.219	.341
Sex, Female	0.108 (−0.154–0.371)	0.194	.399
Amblyopic eye, Left	−0.071 (−0.353–0.210)	−0.121	.601
Amblyopic eye visual acuity	−0.045 (−2.288–2.199)	−0.010	.967
Amblyopic eye group, B&C	−0.070 (−0.364–0.224)	−0.114	.624
ONE (SWE value), amblyopic eye	−0.009 (−0.068–0.050)	−0.072	.756

Dependent variable: Difference in optic nerve sheath diameter between healthy and amblyopic eyes.

CI = confidence interval, ONE = optic nerve elasticity, ONSD = optic nerve sheath diameter, SWE = shear-wave elastography.

## 5. Discussion

Current methods for the assessment and classification of amblyopia remain limited, representing an important challenge given the variable therapeutic success rates reported in these patients.^[1]^ Consequently, there is ongoing interest in identifying imaging-based markers that may provide additional insight into the underlying mechanisms of amblyopia.

In the present study, we evaluated potential structural changes associated with amblyopia using ultrasonographic optic nerve measurements. We found that mean ONSD was significantly lower in amblyopic eyes than in healthy fellow eyes, whereas median ONE values were similar between the 2 groups. Although reduced ONSD may represent a structural feature associated with amblyopia, its biological and clinical significance remains uncertain.

Several studies investigating different components of the visual pathway have explored the pathogenesis of amblyopia.^[2,8,12,14,31]^ Structural and functional alterations have been reported in the lateral geniculate nucleus and visual cortex,^[1,8]^ and more recent studies have also examined anterior visual pathway structures, including the optic nerve, optic disc, retina, and choroid.

In the present study, mean ONSD was significantly lower in amblyopic eyes than in healthy fellow eyes; however, univariable regression analysis demonstrated no significant association between ONSD differences and the severity of visual impairment. ONSD was evaluated because previous studies have associated this parameter with various visual disorders and functional alterations.^[32]^

Previous studies investigating anterior visual pathway structures in amblyopia have reported inconsistent findings. While some demonstrated increased retinal nerve fiber layer or retinal thickness in amblyopic eyes, others found no significant interocular differences.^[10,11,14–16,31]^ Increased choroidal thickness has also been reported,^[12]^ whereas most studies evaluating optic disc area did not identify significant differences between amblyopic and healthy eyes.^[10,11]^ These discrepancies may reflect differences in imaging techniques, study populations, refractive status, and amblyopia subtypes.

To our knowledge, no previous studies have specifically investigated ONSD in patients with amblyopia. Given the essential role of the optic nerve in visual function, assessment of ONSD may provide additional information regarding structural characteristics associated with amblyopia.

Increased ONSD has previously been associated with elevated intracranial pressure in both pediatric and adult populations and is considered a reliable indirect marker of intracranial hypertension.^[33,34]^ Although these mechanisms are generally associated with increased rather than decreased ONSD, they provide important context for understanding the physiological factors that may influence optic nerve sheath measurements.

In contrast, the reduced ONSD observed in our study is unlikely to be explained by intracranial pressure alterations, as such changes would generally be expected to affect both eyes symmetrically. Reduced ONSD may therefore represent a structural characteristic associated with amblyopia; however, because of the cross-sectional design, it remains unclear whether this finding is causally related to amblyopia or represents an incidental association. The clinical significance and potential reversibility of reduced ONSD also remain uncertain.

Previous studies have reported associations between reduced ONSD and optic nerve atrophy in various pathological conditions.^[35–37]^ However, such interpretations should be approached cautiously in amblyopia. Alternative explanations, including subtle biometric or refractive differences, individual anatomical variation, or developmental structural characteristics unrelated to optic nerve atrophy, may also account for the observed findings. Further prospective studies with larger cohorts are needed to clarify the biological and clinical significance of reduced ONSD in amblyopia.

The optic nerve and optic nerve sheath are passive tissues whose mechanical properties may reflect underlying structural alterations.^[32]^ Therefore, evaluation of ONE may provide additional information regarding tissue characteristics associated with amblyopia. In the present study, although SWE values were higher in amblyopic eyes compared with healthy fellow eyes, this difference did not reach statistical significance.

Although the present study did not demonstrate a significant difference in ONE values between amblyopic and healthy eyes, the relatively small sample size may have limited the ability to detect subtle biomechanical differences. Further prospective studies with larger cohorts are needed to better clarify the potential role of ONE in amblyopia.

This study has several limitations. The relatively small sample size may limit statistical power and generalizability, and the cross-sectional design precludes causal or longitudinal interpretation of the findings. In addition, intra- and inter-observer variability analyses were not performed. Although all examinations were conducted by a single experienced radiologist using a standardized imaging protocol, the operator-dependent nature of ultrasonographic and elastographic measurements should be considered. Furthermore, the lack of blinding during ultrasonographic assessment may have introduced measurement-related bias. Finally, residual confounding related to subtle refractive or biometric variations, including axial length differences, cannot be completely excluded and may have influenced ONSD measurements.

ONSD is a dynamic parameter that may be influenced by intracranial pressure variations.^[38]^ However, because comparisons were performed between amblyopic eyes and healthy fellow eyes within the same individual, this potential source of variability may have been partially minimized.

## 6. Conclusion

In conclusion, mean ONSD values were significantly lower in amblyopic eyes than in healthy fellow eyes, whereas ONE values were similar between the 2 groups. These findings suggest that reduced ONSD may represent a structural feature associated with amblyopia. However, univariable regression analysis did not demonstrate a significant association between ONSD difference and the severity of visual impairment, and the biological and clinical significance of reduced ONSD remains uncertain. Given the cross-sectional design and relatively small sample size, further prospective studies with larger cohorts and longitudinal follow-up are needed to better clarify the roles of ONSD and ONE in amblyopia.

## Author contributions

**Conceptualization:** Behice Kaniye Yilmaz, Aynur Diracoglu, Tuba Selcuk Can.

**Data curation:** Behice Kaniye Yilmaz, Aynur Diracoglu, Sevim Ozdemir.

**Investigation:** Behice Kaniye Yilmaz, Aynur Diracoglu, Sevim Ozdemir.

**Methodology:** Behice Kaniye Yilmaz, Aynur Diracoglu, Sevim Ozdemir, Tuba Selcuk Can.

**Resources:** Sevim Ozdemir, Tuba Selcuk Can.

**Supervision:** Aynur Diracoglu, Tuba Selcuk Can.

**Writing** – **original draft:** Behice Kaniye Yilmaz.

**Writing** – **review & editing:** Behice Kaniye Yilmaz.
